# A randomized controlled trial to evaluate the acceptability and effectiveness of two eating disorders prevention interventions: the HEIDI BP-HW project

**DOI:** 10.1186/s12905-023-02607-6

**Published:** 2023-08-23

**Authors:** Isabelle Carrard, Sezen Cekic, Sophie Bucher Della Torre

**Affiliations:** 1 Department of Nutrition and dietetics, Geneva School of Health Sciences, HES-SO University of Applied Sciences and Arts Western Switzerland, Geneva, Switzerland; 2 Department of Psychology, University of Geneva, Geneva, Switzerland

**Keywords:** Eating disorders, Prevention, Body dissatisfaction, Cognitive dissonance, Healthy lifestyle

## Abstract

**Background:**

Eating disorders (ED) are common in Switzerland, as in other Western countries, with a prevalence of any ED of 3.5%. However, no specific prevention intervention has been evaluated in the French-speaking part of the country. In this study, we assessed the acceptability and effectiveness of two well-validated eating disorders prevention interventions: the Body Project intervention (BP), based on cognitive dissonance techniques, and the Healthy Weight intervention (HW), based on the implementation of a healthy lifestyle.

**Methods:**

Forty female students, aged 18–28, with body dissatisfaction, were randomized into three arms: a BP group, an HW group, and a waiting-list control group (WLCG). The primary outcome measure was body dissatisfaction. Secondary outcomes were thin-ideal internalization, dietary restraint, negative affect, and ED psychopathology. Thirty-three participants completed the assessments before and after the one-month interventions or waiting period. A follow-up measurement was conducted one month after the interventions to assess the stability of the results.

**Results:**

Both interventions, delivered via a virtual web platform, were considered acceptable. The reduction in body dissatisfaction was greater in the BP group (r = 0.7; p < 0.01) or the HW group (r = 0.6; p < 0.01) than in the WLCG, with large effect sizes. Dietary restraint and shape concern were also significantly reduced in the BP group (r = 0.6 and r = 0.7, respectively; p < 0.01) and HW group (r = 0.5 and r = 0.5, respectively; p < 0.05) compared to the WLCG, with moderate to large effect sizes. The results obtained in each intervention group were stable at the one-month follow-up.

**Conclusions:**

This study showed encouraging results in young women with body dissatisfaction, arguing in favor of the French adaptations of the BP and HW interventions. However, the feasibility of recruitment was difficult, partly due to the pandemic situation at the time of the study, and should be further considered to improve dissemination.

**Trial registration:**

ClinicalTrials.gov Identifier: NCT04558073, 22/09/2020 and Swiss National Clinical Trial Portal (SNCTP000003978).

**Supplementary Information:**

The online version contains supplementary material available at 10.1186/s12905-023-02607-6.

## Introduction

Because of the serious physical and psychological impairment caused by clinical and subclinical eating disorders, and considering the burden of these disorders and the limits in treatment provision, scholars have called for the development and validation of effective prevention interventions [1]. Eating disorders are common among women, with a peak onset age ranging from 16 to 20 [2]. High rates of somatic and psychiatric comorbidities are associated with eating disorders, such as anxiety, mood disorders, substance use, self-injury, suicide attempts [3, 4], and risks of premature death [5]. However, for various reasons such as shame, stigma, denial, and lack of insurance or facilities providing evidence-based treatment nearby, the rate of people seeking help or benefiting from effective treatment remains low [6–8]. Therefore, the development of effective prevention interventions for eating disorders is a public health priority [9].

A turning point occurred in the field of eating disorders prevention in the 2000s when new interventions targeting risk factors were developed and evaluated. These interventions relied on models such as the Tripartite model formulated by Thompson et al. [10] or the dual-pathway model of bulimic pathology tested by Stice [11], which highlighted the pathways leading to the onset of eating disorders. These models include factors such as thin-ideal internalization, body dissatisfaction, and dieting, indicating target risk factors that can be addressed by eating disorder prevention interventions. In 2016 and 2017, two systematic literature reviews, one of which included a meta-analysis, were able to identify prevention interventions that had shown an effect on specific risk factors with moderate to large effect sizes [12, 13]. When classifying the efficacy of these interventions by levels of prevention [14], media literacy appeared to be effective in reducing risk factors in universal prevention, designed for anyone without considering the presence or absence of any risk factors. Cognitive dissonance and a healthy weight intervention appeared to be effective in reducing risk factors in selective prevention designed for persons with at least one risk factor. Finally, cognitive and behavioral therapy appeared to be effective in reducing risk factors for indicated prevention, designed for persons showing signs or symptoms of pathology [13]. The strongest effects were observed for selective prevention. These findings were recently confirmed in a meta-analytic review, which highlighted that cognitive dissonance and lifestyle intervention were the only interventions targeting risk factors that had the potential to show a real preventive effect, with a 54–77% reduction in future onset of eating disorders, leading to the conclusion that they should be widely implemented [15].

The principles of cognitive dissonance were coined by Festinger [16]. This theory states that when people act in a divergent way from their beliefs, they will feel an uncomfortable feeling (the “dissonance”) that they will try to reduce by changing their acts or beliefs. Cognitive dissonance is the main active principle used in the Body Project intervention (BP), first designed by Stice et al. [17]. In this intervention, participants are required to act deliberately against the beauty ideal standards in a series of exercises, which produces cognitive dissonance, and consequently a change in their beliefs and beauty standards internalization. The BP is the eating disorder prevention intervention that has shown the largest scalability to date, reaching up to 3.5 million girls and young women in 125 countries [18]. In 2019, a meta-analysis of 56 studies that evaluated 68 cognitive dissonance-based eating disorders prevention interventions showed that in comparison with a control group with minimal intervention, cognitive dissonance interventions led to reductions in thin-ideal internalization and body dissatisfaction with moderate effect sizes, and reductions in dieting, negative affect, and eating disorder symptoms with small effect sizes [19]. The BP proved adaptable to various cultures and was delivered with success in countries other than the US, such as Brazil, Sweden, and China [20–22]. Various delivery modalities were also tested, and when clinician-led, peer-led, and internet-delivered versions were compared to an educational video control group, every BP modality showed larger reductions in risk factors than the control group [23]. However, the peer-led group performed better than the other two conditions regarding eating disorder onset over the 4-year follow-up.

The Healthy Weight intervention (HW), which was the second most effective intervention for selective prevention alongside the BP, was at the start a control condition for the latter, designed by Stice et al. [17]. It is based on social psychology and psychoeducation. Participants are encouraged to implement small lifestyle changes in their eating and exercise habits to enhance their health and maintain their weight. They benefit from the group to obtain support and discuss the potential pitfalls. The HW showed similar reductions in eating disorder risk factors as the BP [24, 25]. Moreover, the HW showed higher acceptance than the BP when delivered to athletes [26]. A nutrition-oriented intervention made more sense for athletes preoccupied with performance and health rather than an intervention that discussed the pressure of beauty standards. Other research groups have tested this intervention. A recent study has confirmed the relevance of the HW, renamed the “healthy living intervention”, for ballet dancers [27]. The intervention was renamed because the terminology around weight has changed in the eating disorders field and society and because the intervention did not focus on weight maintenance in the same way as the original HW. Significant reductions in body dissatisfaction, dietary restraint, and eating disorder psychopathology were observed post-intervention compared to a control group. However, this preliminary study in this population of ballet dancers also highlighted that feasibility was difficult, with low rates of recruitment, albeit an obvious need in this population, who report a high level of body dissatisfaction.

In Switzerland, a population-based survey carried out in 2010 of 10’038 residents aged 15–60 years showed prevalence rates of clinical and subclinical eating disorders similar to those observed in other Western countries [28]. The lifetime prevalence of any ED was 3.5%. Among women, lifetime prevalence estimates for any eating disorder, subthreshold binge eating disorder, and any binge eating were 5.3, 0.9, and 5.3%, respectively; among men, they were 1.5, 1.6, and 2.9%, respectively, with 67.9% of women and 49.4% of men reporting seeking treatment for their condition. To our knowledge, no studies assessing eating disorder prevention interventions have been conducted in Switzerland, although prevalence figures demonstrate a need in this domain. Development of new programs has been discouraged because of the numerous existing interventions, most of which have been evaluated only once [29]. Moreover, there is strong evidence that the BP and the HW influence eating disorder risk factors. Therefore, we chose to focus on these two highly validated prevention interventions and to conduct a study with French-speaking female students presenting with body dissatisfaction, a population that has been typically targeted in studies evaluating these interventions, particularly effective for selected prevention. We wanted to evaluate both interventions, because studies have shown that the BP and the HW may have different effects depending on the sub-group [26]. Even though we thought that their influence on the outcome variables would be equivalent in the present study, we wanted to evaluate both interventions in French-speaking Switzerland as a basis for further studies.

The first goal of the current study was to evaluate the acceptability and feasibility of the BP and HW in a population of French-speaking female students in Switzerland aged 18–25. The second goal was to preliminarily evaluate the effectiveness of both interventions in comparison with a waiting-list control group (WLCG) in reducing body dissatisfaction, thin-ideal internalization, dietary restraint, negative affect, and eating disorder psychopathology immediately after intervention, with a randomized controlled study. Third, we wanted to assess whether the observed effects were maintained after a one-month follow-up.

The three hypotheses were that first, the acceptability of both interventions, as assessed by the number of sessions attended by the participants and their satisfaction with the intervention, would be good for both interventions. Second, the BP and HW would improve body dissatisfaction (primary outcome) as well as thin-ideal internalization, dietary restraint, negative affect, and eating disorder psychopathology (secondary outcomes) compared to the WLCG. Third, the post-intervention scores observed in the intervention groups (BP and HW) would be maintained after one month of follow-up.

## Methods

This study was approved by the Ethics Committee of Geneva, Switzerland (project ID 2020 − 01010). The study protocol has been previously published [30]. The HEIDI BP-HW project began in March 2020. The sanitary situation caused by the SARS-CoV-2 pandemic forced us to adapt recruitment methods, the data collection process, and the intervention delivery method. Recruitment and data collection started in January 2021 and ended in May 2022. Due to the health situation, data were collected online, and interventions were conducted via a virtual web platform in small groups of a maximum of six participants to ensure interactivity.

### Participants

Female students from French-speaking Swiss universities were included. More precisely, the inclusion criteria were as follows: to be a female student; to be between 18 and 25 years old with the rationale that the groups would be more homogeneous in their interests and references to beauty ideals (this criterion was the one announced, but we included two older participants, aged 26 and 28, no participant was excluded on the basis of age before the change); to have a body mass index (BMI) between 18.5 and 30 kg/m^2^; be French-speaking (or understand French sufficiently to participate in the intervention) and have lived in Switzerland for at least six months; suffer from body dissatisfaction with a score of at least 26 on the BSQ-8 C [31], which represents moderate body image concerns; and agree to use of a collaborative platform to participate in the intervention group, which implies that her name will be revealed and that the sessions will be recorded. Exclusion criteria were as follows: current eating disorder according to the DSM-5 diagnostic criteria (a past disorder was initially defined as an exclusion criterion, but was revised due to recruitment difficulties, as was the age criterion); current diagnosis of a mood or anxiety disorder; pregnancy.

Forty participants were included in the randomization process. Participants were randomized to one of three groups: BP group (n = 14), HW group (n = 14), and WLCG (n = 12).

.

### Recruitment

Announcements regarding the study were difficult to convey because the students were partially at home during the recruitment period. For this reason, we published a promotional video on the School of Health Sciences Internet website, a link to the study on the website of the University of Geneva, and regular posts on social networks, including paid advertising on Facebook for two months. Health partners shared the information with their network. Because recruitment was slower than expected, we also asked the various schools of the University of Applied Sciences Western Switzerland (HES-SO) to send direct e-mails to their students. A few of them accepted. Finally, when students returned on-site in the fall of 2021, we held announcements on noticeboards, booths, and printed flyers. There was no compensation to participate in the study, neither money nor course credit.

### Procedure and randomization

The persons interested in the study wrote to the project e-mail address and were then contacted by phone by the first author, who checked the inclusion and exclusion criteria and provided detailed information regarding the study. To check the inclusion and exclusion criteria, the first author asked the eight questions of the *Body Shape Questionnaire* (BSQ-8 C) orally, and the person had to answer on the suggested scale from 1 *never* to 6 *always.* The total score was calculated to verify that the score had reached 26. This verbal questioning was used for recruitment purposes only, and if included, the participant completed the BSQ-8 C again before the start of the study. The first author, who is a psychotherapist with clinical experience, asked the person if she had ever been diagnosed with a mood, anxiety, or eating disorder, and if she had ever received psychotherapy, and decided on inclusion based on this discussion. If the participant met the criteria and was interested, she received the information and consent forms by post mail, which she had to return with a signature to be randomized in one of the three arms of the study, with a 1: 1: 1 allocation ratio. Randomization was blocked to ensure that groups of six participants in each arm were regularly formed. The blocks were of variable sizes to protect concealment. The randomization sequence was implemented in the REDCap software (Research Electronic Data Capture; [32, 33]), so that the allocation process was automated.

### Interventions and assessments

The participants were informed of the intervention they were receiving. Participants completed online questionnaires at three time points: (1) before the start of the intervention or the waiting period, (2) four weeks later at the end of the intervention or waiting period, and (3) one month later.

The participants in the BP group received the intervention in four weekly sessions of 90 min, scheduled at the end of the day after school. Two facilitators, the first author, who is a psychologist and an assistant who is a dietitian of a similar age to the participants, led the groups via Teams®, a virtual platform widely used for teaching activities during the 2020 lockdown. We used a French translation of the BP script. The team was trained by the translator of the manual, a researcher at the University of Grenoble-Alpes (France). During the four weeks of intervention, participants were encouraged with discussions and exercises to behave and speak against the current beauty ideals, such as thinness and muscularity, to create cognitive dissonance and distance them from their ideals.

The participants in the HW also received the intervention in four weekly sessions of 90 min, scheduled at the end of the day after school. Two facilitators, the third author, who is a dietitian and an assistant, also a dietitian, with a similar age to the participants, led the groups via Teams®. We translated the script following the original framework, which includes psychoeducation behavioral change techniques, to encourage participants to implement small changes in their lifestyle for health and well-being, as well as group discussions on the encountered difficulties. However, the advice given as a source of inspiration to choose which changes to implement to aim for a health ideal instead of a thin ideal was adapted to the Swiss recommendations for a balanced diet and physical activity. These recommendations included four parts: what, how much, when and how. The advice was given on the quality (what) and quantity (how much) of food and physical activity that had to be aimed for. “When” included advice regarding the structure of the day, and “how” recommended relying on internal cues and feelings to reconnect with food intake and physical activity that would be satisfying and positive for health, without focusing on weight.

The participants in the WLCG had to complete two assessments, each separated by a one-month interval, during which they received no intervention. They then received the BP, because that intervention showed the largest effects [24, 25]. They completed the questionnaires at the end of the BP, but these measures were not used in the present study.

### Measures

Participants completed questionnaires that were implemented in the REDCap software before the intervention or the waiting list (pre), after the intervention or the waiting list (post), and one month later (follow-up).

#### Primary outcome

**Body dissatisfaction** was assessed using a short version of the *Body Shape Questionnaire* (BSQ-8 C) [31] which includes eight items selected from the original 34-item Body Shape Questionnaire [34], to assess preoccupations with body shape encountered in eating disorders. The items are rated on a 6-point Likert scale ranging from 1 *never* to 6 *always*. The 34-item version of the BSQ has been validated in French [35]. Cronbach’s alpha for the BSQ in this study was 0.77, indicating satisfactory internal consistency [36].

#### Secondary outcomes

**Thin-ideal internalization** was assessed using the *Socio-Cultural Attitudes Towards Appearance Questionnaire* (SATAQ-4; [37]), which includes five items evaluating the desire to look thin and lean. Items are rated on a 5-point Likert scale ranging from 1 *strongly disagree* to 5 *strongly agree*. The SATAQ-4 has been translated and validated in French [38]. Cronbach’s alpha for the SATAQ-4 thin-ideal internalization subscale in this study was 0.81, indicating satisfactory internal consistency.

**Dietary restraint** was assessed using the Restrained eating subscale of the *Dutch Eating Behaviour Questionnaire* (DEBQ; [39]). This subscale includes ten questions that evaluate whether food intake is restrained to monitor weight. The items are rated on a scale of 1 *never* to 5 *always*. The DEBQ has been translated and validated in French [40]. Cronbach’s alpha for the DEBQ dietary restraint subscale in this study was 0.87, indicating satisfactory internal consistency.

**Negative affect** was assessed using the *Hospital Anxiety and Depression Scale* (HAD; [41]) composed of seven items assessing anxiety (Cronbach’s alpha = 0.70) and seven items assessing depression (Cronbach’s alpha = 0.68). Items are rated on a scale of 0 *never* to 3 *very often*. The HAD was translated and validated in French [42, 43]. The internal consistency of the depression subscale was less than 0.70, which is considered unsatisfactory.

**Eating disorder psychopathology** was assessed using the *Eating Disorder Examination-Questionnaire* (EDE-Q; [44]) which includes 28 items assessing four core dimensions of eating disorders: dietary restraint (Cronbach’s alpha = 0.76), eating concern (Cronbach’s alpha = 0.74), shape concern (Cronbach’s alpha = 0.86), and weight concern (Cronbach’s alpha = 0.72), and a total score (Cronbach’s alpha = 0.92), which is an indicator of psychopathology on a continuum. Subscale items are rated on a scale of 0 to 6, indicating severity *(Not at all* to *Markedly*) of frequency (*No days* to *Every day*). The EDE-Q has been translated and validated in French [45]. Cronbach’s alphas showed satisfactory internal consistency.

#### Sociodemographic variables

Self-reported weight and height were used to calculate participants’ BMI. Self-reported weight and height have been shown to correlate strongly with anthropometric measurements [46]. Questions about current age and country of origin (Switzerland, Europe, the United States or other Anglo-Saxon countries, other) were also included in REDCap and used to describe the population.

#### Satisfaction

Post-intervention, the participants had to assess their satisfaction with the intervention using four questions rated on a Likert scale of 0 *not at all* to 5 *absolutely*. These four questions assessed the usefulness of the interventions and their helpfulness, whether the concepts were understandable, and whether the exercises were useful.

### Statistical analysis

The first hypothesis, stating that both interventions would be judged acceptable, was tested by calculating the number of sessions attended by the participants in each intervention and the scores on the satisfaction subscales. We also compared the attendance at each intervention and calculated t-tests between the BP group and the HW group to compare the mean scores on the questions of satisfaction.

We considered the number of persons who dropped out (i.e. those who did not start the intervention after being randomized or who did not complete the second assessment after having followed one or more sessions of one of the interventions) as an index of feasibility. The BP and HW groups were compared using the chi-squared test. An attrition rate of 10%, found in previous studies with the same interventions [17] was used as a reference.

**Fig. 1:**
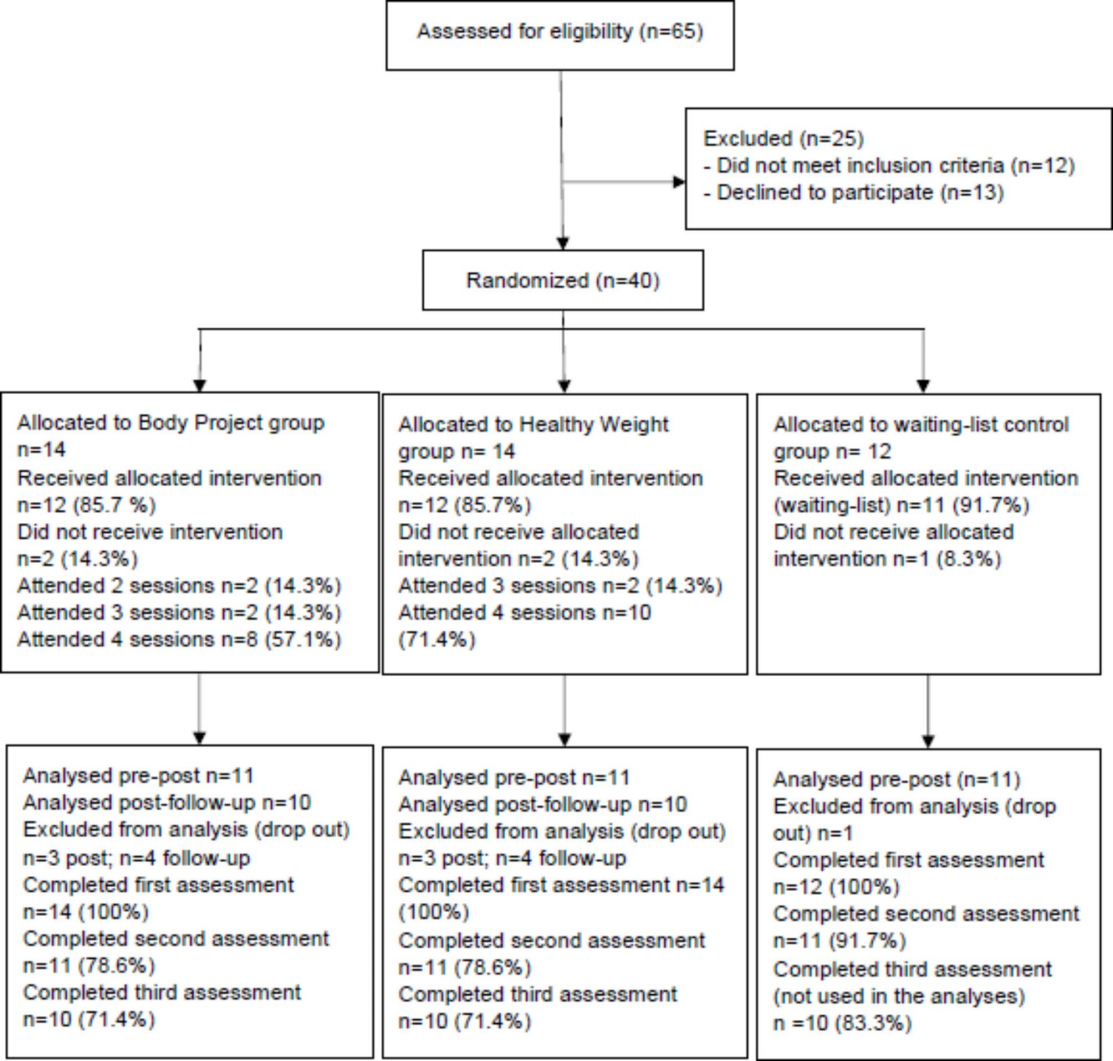
Participants’ flow diagram throughout the study

The second hypothesis, that the BP and the HW would improve body dissatisfaction, thin-ideal internalization, dietary restraint, negative affect, and eating disorder psychopathology compared to the WLCG, was tested using per-protocol analyses with a sample of 33 participants. A sample of 90 participants was estimated to be necessary to detect group differences with large effect sizes [30]. However, as depicted in Fig. 1, which shows the flow of participants during the study, only 40 participants were included in the randomization. Because of the small sample size and unusual conditions under which this preliminary study was conducted, we decided to include only participants who completed both the first and second assessments in our analyses (per-protocol analyses) and to perform non-parametric Wilcoxon rank tests. This deviation from the analysis plan announced in the protocol, which included ANCOVA, was suggested by the statistician involved in the study at the data analysis stage and discussed with the entire team. This suggestion made it possible to answer the research hypotheses with more robust analyses that were also straightforward to interpret. The decision to perform a per-protocol analysis was privileged to avoid raw data manipulation. A comparison between completers and dropouts was performed using t-tests and is presented in Supplementary Table 1 (Additional File 1). No differences were found between the completers and dropouts. Supplementary Table 2 (Additional File 2) shows the pre-post within-group analyses for comparison with the initial power analysis.

The differences in primary and secondary outcomes between pre- and post-assessments for each participant were calculated and then, group comparisons of the difference scores were performed: BP group versus WLCG and HW group versus WLCG, with non-parametric Wilcoxon rank tests. Wilcoxon tests were preferred over standard t-tests to control for departure from normality and homoscedasticity. Due to our small sample size, we decided to interpret our results in terms of effect sizes and not only in terms of *p*-values, which are very sensitive to N. p-values are nevertheless reported in tables and results description.

To assess the effect sizes of between-group comparisons, the Wilcoxon effect size (*r*) was calculated together with bootstrap effect size confidence intervals. The interpretation values for the Wilcoxon effect sizes (*r*) are 0.1 - < 0.3, small; 0.3 - < 0.05, moderate; and ≥ 0.05, large [47]. Effect sizes obtained for each group comparison (BP group versus WLCG and HW group versus WLCG) on each primary and secondary outcome, were compared to quantify which one of both interventions had the greatest effect compared to the control group.

The third hypothesis, stating that the effects observed in the intervention groups would be maintained, was tested with non-parametric paired Wilcoxon rank tests, with a systematic comparison of the primary and secondary outcome scores obtained post-intervention and after a one-month follow-up for both interventions. Non-significant differences with small effect sizes were deemed representative of the stability of previously observed effects.

Statistical analyses were conducted using IBM SPSS 26 and R version 4.1.2 (2021-11-01).

## Results

### Participants’ characteristics

The mean age of participants was 22.3 years old (± 2.0, range 19–28), and the mean BMI was 22.5 (± 2.3, range 18.8–27.6). Thirty-one participants (77.5%) were Swiss, seven (17.5%) came from other European countries, and two (5%) were from outside Europe. Five (12.5%) participants declared that they had suffered from an eating disorder in the past and that their eating behavior normalized, but their body dissatisfaction did not.

### Acceptability and feasibility

A comparison of the number of attended sessions among the participants allocated to each intervention (n = 14 in the BP group and n = 14 in the HW group) is shown in Fig. 1. Participants were more committed to the HW than to the BP, with 71.4% of participants attending the four sessions of the HW and 57.1% of participants attending the four sessions of the BP. Figure 1 also shows that 13 women (20% of those whose eligibility was assessed) withdrew from enrollment during the recruitment process. No explanation could be obtained for these withdrawals, as they did not respond to our emails, phone calls, or mail.

In terms of satisfaction, each mean score on the satisfaction subscales was equal to or greater than 4 on a scale of 1 to 5 in both interventions, indicating good satisfaction with various aspects of the interventions. No significant differences emerged between the interventions in the comparisons between satisfaction subscale scores. Table 1 shows that the mean scores obtained for the four items of satisfaction were comparable for both interventions, except for one participant who judged the exercises to be less useful for the HW, decreasing the mean score of the exercises’ usefulness for this group. This participant mentioned in the comments that she had already followed nutritional treatment and therefore knew well the techniques that were trained in the program to implement lifestyle changes.

**Table 1:** Means, standard deviations (SD), and comparisons of satisfaction items between interventions

	Body Project (n = 11)		Healthy Weight (n = 11)		t-test
	Mean (SD)	Min-Max	Mean (SD)	Min-Max	p-value
Intervention useful	4.6 (0.5)	4–5	4.5 (0.8)	3–5	0.760
Intervention helpful	4.0 (0.8)	2–5	4.1 (0.8)	3–5	0.793
Easy to understand	5.0 (0.0)	5–5	4.8 (0.4)	4–5	0.167
Exercises useful	4.6 (0.5)	4–5	4.2 (1.2)	2–5	0.250

Regarding feasibility, the number of participants who did not start the intervention was comparable between the BP and HW groups (n = 2 in BP and n = 2 in HW, p = 1.000). The reasons for withdrawal were unknown, except for one participant who reported schedule constraints. In the BP, a third participant who had followed two sessions did not complete the second assessment, and in the HW, a third participant who had followed the four sessions did not complete the second assessment.

Unhealthy attitudes were monitored during the interventions by the two leaders of the group sessions to prevent interventions from harming the participants. None of the participants had to be excluded because of any harmful effects.

### Interventions effects

Table 2 shows pre and post-intervention raw scores for both the intervention groups and the control group. Raw mean scores of the primary and secondary outcomes are reported, namely body dissatisfaction, thin-ideal internalization, dietary restraint, negative affect and eating disorders psychopathology for the 33 participants who completed the first and second assessments. Differences between pre and post scores were then calculated within each group, and these pre-post difference scores were compared with Wilcoxon rank tests between each intervention group and the WLCG. Effect sizes’ confidence intervals and *p*-values for pre-post differences group comparisons are displayed in Table 3.

**Table 2:** Summary of raw means (M) and standard deviations (SD) for the three groups: Body Project intervention (BP), Healthy Weight intervention (HW) and waiting-list control group (WLCG)

	BP (n = 11)		HW (n = 11)		WLCG (n = 11)	
	M(SD) pre	M(SD) post	M(SD) pre	M(SD) post	M(SD) pre	M(SD) post
BSQ Body dissatisfaction	27.9 (5.2)	18.3 (4.9)	30.5 (7.5)	21.5 (5.1)	26.3 (5.7)	24.0 (5.0)
SATAQ-4 Thin-ideal internalization	3.4 (0.9)	2.8 (0.7)	3.3 (0.9)	2.9 (0.9)	3.1 (1.1)	3.1 (0.9)
DEBQ Dietary restraint	3.1 (0.7)	2.6 (0.9)	3.1 (0.8)	2.6 (0.8)	2.6 (0.9)	2.7 (0.8)
HAD Anxiety	1.7 (0.4)	1.4 (0.4)	1.4 (0.5)	1.1 (0.5)	1.2 (0.5)	1.1 (0.5)
HAD Depression	0.8 (0.6)	0.8 (0.4)	0.7 (0.5)	0.6 (0.4)	0.6 (0.2)	0.8 (0.4)
EDE-Q Dietary restraint	1.4 (1.1)	0.6 (0.6)	1.7 (1.0)	1.2 (0.8)	0.9 (1.2)	1.2 (1.3)
EDE-Q Eating concern	1.5 (1.1)	0.7 (0.7)	1.7 (1.0)	1.1 (0.8)	1.3 (1.0)	0.9 (0.9)
EDE-Q Shape concern	3.6 (1.4)	1.7 (1.0)	3.3 (1.4)	2.3 (1.2)	3.1 (1.1)	3.0 (1.0)
EDE-Q Weight concern	3.1 (1.3)	1.9 (1.0)	2.8 (1.5)	2.1 (1.3)	2.9 (1.2)	2.7 (1.1)
EDE-Q ED psychopathology	2.4 (1.1)	1.2 (0.7)	2.4 (1.0)	1.7 (0.8)	2.1 (0.9)	1.9 (0.9)
Body Mass Index	22.9 (2.7)	22.8 (3.1)	22.2 (2.2)	22.3 (2.2)	22.5 (2.0)	22.2 (2.3)

Note. BSQ Body Shape Questionnaire; DEBQ Dutch Eating Behavior Questionnaire; ED eating disorders; EDE-Q Eating Disorder Examination-Questionnaire; HAD Hospital Anxiety and Depression Scale; SATAQ-4 Socio-Cultural Attitudes Towards Appearance Questionnaire

**Table 3:** Effect sizes (r), confidence intervals (CI), and significance of the comparisons of pre-post scores differences between the Body Project intervention (BP) or the Healthy Weight intervention (HW) and the waiting-list control group (WLCG)

	BP-WLCG differences (n = 11 per group)			HW-WLCG differences (n = 11 per group)		
	r	CI (95%)	Effect size	r	CI (95%)	Effect size
BSQ Body dissatisfaction	0.7**	0.4–0.8	Large	0.6**	0.2–0.8	Large
SATAQ-4 Thin-ideal internalization	0.3	0.01–0.7	Moderate	0.2	0.01–0.6	Small
DEBQ Dietary restraint	0.6**	0.2–0.8	Large	0.5 *	0.04–0.8	Moderate
HAD Anxiety	0.2	0.01–0.5	Small	0.1	0.00–0.4	Small
HAD Depression	0.3	0.01–0.6	Small	0.5*	0.1–0.7	Moderate
EDE-Q Dietary restraint	0.7***	0.5–0.8	Large	0.3	0.01–0.7	Moderate
EDE-Q Eating concern	0.3	0.02–0.7	Moderate	0.2	0.01–0.6	Small
EDE-Q Shape concern	0.7**	0.4–0.8	Large	0.5*	0.1–0.7	Moderate
EDE-Q Weight concern	0.4	0.04–0.7	Moderate	0.2	0.01–0.5	Small
EDE-Q ED psychopathology	0.7**	0.3–0.8	Large	0.3	0.01–0.7	Moderate
Body Mass Index	0.01	0.00–0.02	Small	0.4	0.02–0.7	Moderate

Note. BSQ Body Shape Questionnaire; DEBQ Dutch Eating Behavior Questionnaire; ED eating disorders; EDE-Q Eating Disorder Examination-Questionnaire; HAD Hospital Anxiety and Depression Scale; SATAQ-4 Socio-Cultural Attitudes Towards Appearance Questionnaire

* p < .05; ** p < .01; *** p < .001

Pre-post difference scores for body dissatisfaction assessed with the BSQ, dietary restraint assessed with the DEBQ, dietary restraint assessed with the EDE-Q, shape concern, and eating disorder psychopathology assessed with the EDE-Q were significantly *greater* in the BP group than in the WLCG group (in absolute value), with large effect sizes (Table 3). Pre-post difference scores for thin-ideal internalization assessed with the SATAQ-4, eating concern, and weight concern assessed with the EDE-Q were *greater* for the BP group than for the WLCG group (in absolute value), with moderate effect sizes, but here, the Wilcoxon tests did not reach significance.

The pre-post difference scores for body dissatisfaction assessed with the BSQ were significantly *greater* in the HW group than in the WLCG, with a large effect size (Table 3). Pre-post difference scores for dietary restraint assessed with the DEBQ, for depression assessed with the HAD, and for shape concern and eating disorder psychopathology assessed with the EDE-Q were *greater* in the HW group than in the WLCG, with moderate effect sizes. Pre-post difference scores for dietary restraint assessed with the EDE-Q, eating disorder psychopathology assessed with the EDE-Q, and BMI were *greater* in the HW group than in the WLCG, with moderate effect sizes. However, the Wilcoxon test did not reach statistical significance.

**Fig. 2:**
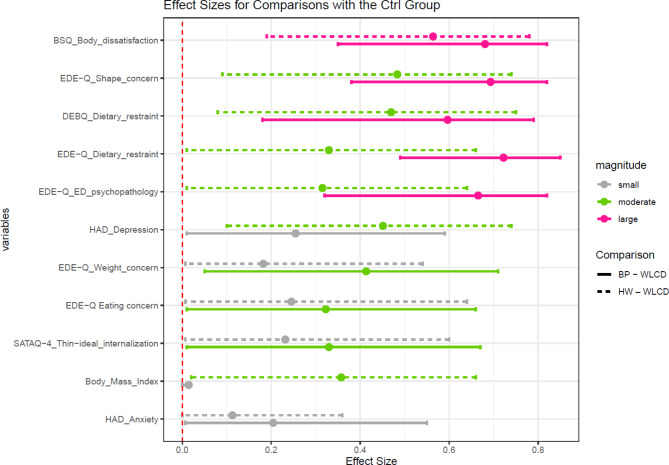
Effect sizes for the pre-post differences scores comparisons between each intervention (BP Body Project and HW Healthy Weight) with the waiting-list control group *Note*. BSQ Body Shape Questionnaire; DEBQ Dutch Eating Behavior Questionnaire; ED eating disorders; EDE-Q Eating Disorder Examination-Questionnaire; HAD Hospital Anxiety and Depression Scale; SATAQ-4 Socio-Cultural Attitudes Towards Appearance Questionnaire

A graphical juxtaposition of all effect sizes obtained for these pre-post scores differences in group comparisons between both interventions and the WLCG was carried out (Fig. 2). The results revealed that the effects obtained with the BP were generally larger than those obtained with the HW, except for depression assessed with the HAD, for which the group comparison between HW and WLCG provided a moderate effect size and the group comparison between BP and WLCG had a small effect size.

### Follow-up stability

Finally, Wilcoxon rank tests were used to assess whether the post-intervention scores were stable at follow-up within each intervention. After screening all primary and secondary outcomes, we found no significant differences between post- and follow-up scores, and effect sizes were negligible for each outcome in both intervention groups (Table 4), revealing stability between post-intervention and follow-up scores one month later.

**Table 4:** Effect sizes (r), confidence intervals (CI), and significance of the comparisons between scores obtained post-intervention and at follow-up (Wilcoxon rank tests) in the Body Project Intervention (BP) and in the Healthy Weight Intervention (HW)

	BP post-follow-up differences (n = 10)			HW post-follow-up differences (n = 10)		
	r	CI (95%)	Effect size	r	CI (95%)	Effect size
BSQ Body dissatisfaction	0.2	0.01–0.6	Small	0	0–0	Small
SATAQ-4 Thin-ideal internalization	0.04	0–0.1	Small	0.2	0–0.5	Small
DEBQ Dietary restraint	0.2	0–0.5	Small	0.02	0–0.04	Small
HAD Anxiety	0.01	0–0.02	Small	0.1	0–0.4	Small
HAD Depression	0.1	0–0.3	Small	0.1	0–0.2	Small
EDE-Q Dietary restraint	0.1	0–0.3	Small	0.1	0–0.3	Small
EDE-Q Eating concern	0.1	0–0.2	Small	0.2	0.01–0.6	Small
EDE-Q Shape concern	0.2	0.01–0.6	Small	0.02	0–0.04	Small
EDE-Q Weight concern	0.2	0–0.5	Small	0.2	0–0.6	Small
EDE-Q ED psychopathology	0.2	0–0.6	Small	0.1	0–0.4	Small
Body Mass Index	0	0–0	Small	0.02	0–0.04	Small

Note. BSQ Body Shape Questionnaire; DEBQ Dutch Eating Behavior Questionnaire; ED eating disorders; EDE-Q Eating Disorder Examination-Questionnaire; HAD Hospital Anxiety and Depression Scale; SATAQ-4 Socio-Cultural Attitudes Towards Appearance Questionnaire

## Discussion

This study, called the HEIDI BP-HW project, evaluated the acceptability and preliminary effectiveness of two well-validated eating disorders prevention interventions, the BP and HW, in the French-speaking region of Switzerland. Overall, our three hypotheses were confirmed: both interventions proved to be acceptable; the primary outcome, which was body dissatisfaction, was reduced after both interventions and some secondary outcomes as well; and the post-intervention scores were stable at a one-month follow-up. However, these positive results have to be tempered by the difficulty of the feasibility of the recruitment. The results of this study should be interpreted with caution because of the small sample size.

Recruitment was complicated by the lockdown and difficulty in reaching our population of interest. Therefore, we used social networks and internet announcements with modest success. The difficulty in reaching and engaging young persons at risk of eating disorders in prevention programs has already been pointed out [48] and did not only depend on the COVID situation, as shown in a study with ballet dancers which obtained a low rate of participation, whereas the target population exhibited high levels of body dissatisfaction [27]. Contrary to the major randomized controlled trials conducted on the efficacy of the BP and HW [24, 25], participants did not receive any compensation to complete the assessments in the present study. This may have carried weight for students who are over-solicited as research subjects. Money, credits, or mandatory requirements should not be underestimated to increase the feasibility of recruitment as a way to thwart the widespread pressure of beauty ideals in society. As theorized by the self-objectification theory, women believe they have to change their bodies to fit social standards, and not that they can fight social pressures to decrease their body dissatisfaction [49]. They do not spontaneously rush to interventions that target body dissatisfaction.

Acceptability, including satisfaction with the BP and the HW, has rarely been assessed and compared between these two interventions. However, a preference for the HW over the BP, both adapted for athletes, has been documented in this population [26]. In the present study, an examination of the answers to the questions on usefulness, understandability, and helpfulness did not reveal any difference between the two interventions. Both can be proposed for a non-specific population and obtain similar satisfaction. Interestingly, the flow diagram also highlighted that a higher percentage of participants among those allocated to the HW attended the entire program. This difference was not observed in previous studies [17, 24]. In each intervention, 14 per cent of the participants did not start the intervention at all and 21 per cent, including those who did not start the intervention and those who did not complete the post-assessment, were considered dropouts and were not analyzed. This rate was higher than the 10 per cent of dropout rate reported by Stice et al. [17]. Only one participant reported conflicting schedules. Body shame, which is associated with body dissatisfaction and is known to prevent people from seeking treatment for eating disorders [7, 50], may be another reason for not daring to start interventions. In most of the studies evaluating the BP or the HW, participants simply reporting body dissatisfaction were enrolled. We selected participants according to a specific score representing at least moderate concern with body image. During the inclusion process, several participants mentioned the difficulty of talking about this topic in a group and fear of starting the intervention. This may have influenced the percentage of participants in this study who dropped out before starting the intervention. This may also explain the percentage of people who withdrew from the enrollment process after contacting us for information about the study.

The BP, which promoted cognitive dissonance in participants, produced significant decreases between pre and post scores in comparison with the WLCG with large effect sizes for body dissatisfaction, shape concern, dietary restraint and eating disorder psychopathology. The post-intervention scores of the BP group were maintained at the one-month follow-up. These results are comparable to those reported by numerous studies evaluating this intervention, in the US and internationally [20, 22, 24]. Contrary to most of these previous interventions, no significant effect could be detected with the BP for group comparisons of a pre-post score of thin-ideal internalization and negative affect, although a difference of moderate effect size was obtained for thin-ideal internalization. The small sample size may be part of the explanation; however, thin-ideal internalization has been seen as the main mediator explaining the effect of the BP [51, 52]. As mentioned by Amaral et al. [53], the effectiveness of the BP across different cultures may be explained by its participant-driven nature. In addition, we could say that the BP also adapts to different times.Thinness was not the main beauty ideal that emerged during the BP sessions. Participants were also concerned with having a toned body, having the right shapes at the right place, and looking “healthy” and “confident”. This may explain why we did not capture a decrease in the thin-ideal internalization score with the SATAQ-4 questionnaire subscale, which focuses on thinness. We should have used a questionnaire that embraces a wider concept of feminine beauty ideals.

The HW, which promoted behavioral techniques to gradually implement new health habits, produced significant group comparisons with the WLCG of pre-post scores with large to moderate effect sizes for body dissatisfaction, shape concern, dietary restraint, and depression. The post-intervention scores of the HW group were maintained at the one-month follow-up. In the same way as previously observed [24], the effect sizes were smaller for some outcomes than for those obtained with the BP. In the present study, however, the group difference between the HW and WLCG for the HAD subscale of the depression score had a moderate effect size, whereas no improvement was observed in the comparison between the BP and WLCG. The feeling of regaining control over one’s lifestyle habits and the positive supportive effect of the group may be one hypothesis explaining why negative affect decreased in the HW in comparison with the WLCG.

In summary, these study results suggest that the French versions of the BP and HW were successful adaptations of the original programs designed for US populations. The French manual of the BP was translated by a team of the University of Grenoble-Alpes in collaboration with the original authors of the BP. Moreover, we were trained by the person who led the translation to ensure that the spirit of the program was preserved. The HW was translated and adapted by us because, to our knowledge, no French version exists. Following the recommendations of Swami and Barron [54] for the cultural adaptations of psychometric tools, we paid attention to the semantic aspects of the manual and cultural adaptation. The structure of the program was kept identical, but the content of the psychoeducational material was adapted to local recommendations for a healthy lifestyle. The results of the present study suggest a suitable adaptation for our population. Finally, because of the protective measures against SARS-COV-2, we provided both interventions to the participants via TEAMS® instead of face-to-face groups as usually carried out. The positive results obtained on several risk factors for eating disorders also highlight that both interventions can be relocated on a virtual platform, as it has already been successfully shown for the BP [22].

The main strength of this study was that it was the first to evaluate the effectiveness of the BP and HW in a French-speaking region of Switzerland, where a prevalence of disordered eating similar to other Western countries has been reported. The study contained three randomized arms, and each intervention was compared with a waiting-list control group. However, this study had several limitations that need to be mentioned. Due to the unique circumstances at the time of this study, recruitment was slower and the number of participants in each arm was smaller than expected. This prompted us to adjust our previously reported inclusion and exclusion criteria. Due to limited funding, we had to close the participants’ inclusion in the study before reaching the sample size estimated as necessary to detect large effect sizes. The participants in the study may have been highly motivated by the social contact provided by the meetings, which may have increased the satisfaction scores for both interventions. The statistical analyses did not follow the announced plan and were conducted per protocol, which may have overestimated the effectiveness of the interventions in real-life settings, and the sample was self-selected. The internal consistency of the rating scales was satisfactory, except for the depression subscale, for which the results should be interpreted with caution. Finally, the stability of the pre-post effects after one month of follow-up could only be evaluated within groups without a comparison with the WLCG.

This study showed preliminary encouraging results on eating disorder risk factors in at-risk young women. The preventive effect of these interventions on eating disorders should be confirmed in large-scale, long-term studies. However, both interventions have already provided numerous proof of their efficacy in primary research, systematic reviews, and meta-analyses [12, 13, 15]. Body dissatisfaction has numerous adverse consequences beyond eating disorders, such as depression [55], and disseminating interventions that directly target this variable is of main interest to public health. Future research should examine which subgroup of the population may be more satisfied with which interventions, and whether male participants can benefit similarly from both interventions. The most important point is how to make these interventions desirable for the target population to ease participant inclusion.

### Electronic supplementary material

Below is the link to the electronic supplementary material.

**Supplementary Table 1**: Summary of raw means (M) and standard deviations (SD) for the three assessment times and comparisons of completers and dropouts using t-tests.

**Supplementary Table 2**: Effect sizes and significance tests for within-group differences pre-post intervention: Body Project intervention (BP), Healthy Weight intervention (HW)

## Data Availability

The datasets used and/or analyzed during the current study are available from the corresponding author upon reasonable request.
