# Supplementary material for: A randomized controlled trial to evaluate the acceptability and effectiveness of two eating disorders prevention interventions: the HEIDI BP-HW project
Source: BMC Womens Health. 2023 Aug 23;23:446. doi: 10.1186/s12905-023-02607-6 (PMC10463671; doi:10.1186/s12905-023-02607-6)
Supplement: Supplementary file 1 — Supplementary Table 1: Summary of raw means (M) and standard deviations (SD) for the three assessment times and comparisons of completers and dropouts using t-tests. [file 12905_2023_2607_MOESM1_ESM.docx]

Supplementary Table 1. Summary of raw means (M) and standard deviations (SD) for the three assessment times and comparisons of completers and dropouts using t-tests.

|  | Pre assessment (n=40) | | | Post assessment (n=33) | | | Follow up | |
| --- | --- | --- | --- | --- | --- | --- | --- | --- |
|  | M(SD) completers at post (n=33) | M(SD) dropout at post (n=7) | t-test p-value | M(SD) completers at follow up  (n=30) | M(SD) dropout at follow up  (n=3) | t-test p-value | M(SD)  (n=30) |  |
| BSQ Body dissatisfaction | 28.2 (6.3) | 28.6 (6.7) | .902 | 21.5 (5.6) | 18.7 (2.5) | .390 | 20.9 (6.6) |  |
| SATAQ-4 Thin-ideal internalization | 3.3 (0.9) | 3.4 (0.6) | .776 | 3.0 (0.8) | 2.4 (0.6) | .255 | 2.7 (0.9) |  |
| DEBQ Dietary restraint | 2.9 (0.8) | 3.3 (1.1) | .252 | 2.6 (0.8) | 2.7 (0.8) | .895 | 2.3 (0.9) |  |
| HAD Anxiety | 1.4 (0.5) | 1.3 (0.4) | .631 | 1.2 (0.5) | 1.4 (0.5) | .401 | 1.2 (0.6) |  |
| HAD Depression | 0.7 (0.5) | 0.6 (0.3) | .698 | 0.7 (0.4) | 0.5 (0.1) | .431 | 0.7 (0.5) |  |
| EDE-Q Dietary restraint | 1.3 (1.1) | 1.3 (1.3) | .951 | 1.0 (1.0) | 0.7 (0.5) | .559 | 1.0 (1.1) |  |
| EDE-Q Eating concern | 1.5 (1.0) | 1.3 (1.0) | .642 | 0.9 (0.8) | 0.9 (0.5) | .957 | 0.8 (1.1) |  |
| EDE-Q Shape concern | 3.3 (1.3) | 3.0 (1.4) | .618 | 2.4 (1.2) | 2.0 (0.4) | .663 | 2.2 (1.3) |  |
| EDE-Q Weight concern | 3.0 (1.4) | 2.3 (1.6) | .217 | 2.2 (1.2) | 2.1 (1.3) | .875 | 1.9 (1.3) |  |
| EDE-Q ED psychopathology | 2.3 (1.0) | 2.0 (1.2) | .489 | 1.6 (0.9) | 1.4 (0.5) | .732 | 1.5 (0.9) |  |
| Body Mass Index | 22.5 (2.3) | 22.1 (2.6) | .641 | 22.3 (2.3) | 23.8 (4.2) | .314 | 22.5 (2.3) |  |

Note. BSQ Body Shape Questionnaire; DEBQ Dutch Eating Behavior Questionnaire; ED eating disorders; EDE-Q Eating Disorder Examination-Questionnaire; HAD Hospital Anxiety and Depression Scale; SATAQ-4 Socio-Cultural Attitudes Towards Appearance Questionnaire.
