# Supplementary material for: A randomized controlled trial to evaluate the acceptability and effectiveness of two eating disorders prevention interventions: the HEIDI BP-HW project
Source: BMC Womens Health. 2023 Aug 23;23:446. doi: 10.1186/s12905-023-02607-6 (PMC10463671; doi:10.1186/s12905-023-02607-6)
Supplement: Supplementary file 2 — Supplementary Table 2: Effect sizes and significance tests for within-group differences pre-post intervention: Body Project intervention (BP), Healthy Weight intervention (HW) [file 12905_2023_2607_MOESM2_ESM.docx]

**Supplementary Table 2** Effect sizes and significance tests for within-group differences pre-post intervention: Body Project intervention (BP), Healthy Weight intervention (HW)

|  | BP (n=11) | | | HW (n=11) | | |
| --- | --- | --- | --- | --- | --- | --- |
|  | p-value | Effect size | Magnitude | p-value | Effect size | Magnitude |
| BSQ Body dissatisfaction | 0.038 | 0.717 | large | 0.041 | 0.596 | large |
| SATAQ-4 Thin-ideal internalization | 0.163 | 0.416 | moderate | 0.616 | 0.296 | small |
| DEBQ Dietary restraint | 0.072 | 0.295 | small | 0.531 | 0.316 | moderate |
| HAD Anxiety | 0.223 | 0.289 | small | 0.616 | 0.239 | small |
| HAD Depression | 1.0 | 0.050 | small | 0.585 | 0.176 | small |
| EDE-Q Dietary restraint | 0.047 | 0.373 | moderate | 0.616 | 0.274 | small |
| EDE-Q Eating concern | 0.047 | 0.394 | moderate | 0.531 | 0.310 | moderate |
| EDE-Q Shape concern | 0.038 | 0.603 | large | 0.08 | 0.365 | moderate |
| EDE-Q Weight concern | 0.047 | 0.494 | moderate | 0.531 | 0.218 | small |
| EDE-Q ED psychopathology | 0.011 | 0.511 | large | 0.220 | 0.350 | moderate |
| Body Mass Index | 1.0 | 0.049 | small | 1.0 | 0.014 | small |

Note. BSQ Body Shape Questionnaire; DEBQ Dutch Eating Behavior Questionnaire; ED eating disorders; EDE-Q Eating Disorder Examination-Questionnaire; HAD Hospital Anxiety and Depression Scale; SATAQ-4 Socio-Cultural Attitudes Towards Appearance Questionnaire.
